# ASO Author Reflections: Understanding the Prognostic Role of Claudin 18.2 in Resected Pancreatic Cancer

**DOI:** 10.1245/s10434-026-20312-0

**Published:** 2026-07-29

**Authors:** Jun Yoshino, Hakon Blomstrand, Sebastian Fjellander, Johan Svensson, Asif Halimi, Ella Kesti, Hanna Seppänen, Caroline Vilhav, Daniel Öhlund, Caj Haglund, Nils O Elander, Peter Naredi, Malin Sund, Oskar Franklin

**Affiliations:** 1 Department of Diagnostics and Intervention, Surgery, Umeå University, Umeå, Sweden; 2 Department of Hepatobiliary and Pancreatic Surgery, Graduate School of Medicine, Institute of Science Tokyo, Tokyo, Japan; 3 Department of Clinical Pathology, and Department of Biomedical and Clinical Sciences, Linköping University, Linköping, Sweden; 4 Department of Statistics, Umeå School of Business Economics and Statistics, Umeå University, Umeå, Sweden; 5 Department of Surgery, University of Helsinki and Helsinki University Hospital, Helsinki, Finland; 6 Department of Surgery, Translational Cancer Medicine Research Program, Faculty of Medicine, University of Helsinki and Helsinki University Hospital, Helsinki, Finland; 7 Department of Transplantation Surgery, Institute of Clinical Sciences, Sahlgrenska Academy, University of Gothenburg and Transplant Institute, Sahlgrenska University Hospital, Gothenburg, Sweden; 8 Department of Diagnostics and Intervention, Oncology, and Wallenberg Centre for Molecular Medicine (WCMM), Umeå University, Umeå, Sweden; 9 Department of Oncology, and Department of Biomedical and Clinical Sciences, Linköping University, Linköping, Sweden

## Past

Despite advances in surgical and systemic therapy, pancreatic ductal adenocarcinoma (PDAC) remains one of the most lethal malignancies.^1^ The success of zolbetuximab as a CLDN18.2‑targeted therapy in gastric cancer has generated considerable interest in its potential application in other types of cancer, such as PDAC.^2^ However, reported rates of CLDN18.2 positivity in PDAC vary widely because of methodological differences in antibodies, scoring criteria, and cohort size.^3,4^ Consequently, the true prevalence and prognostic relevance of CLDN18.2 in resected PDAC remain incompletely defined, highlighting the need for evaluation in larger, multicenter cohorts.

## Present

In our recent multicenter cohort of 599 resected PDACs,^5^ 23% of tumors expressed CLDN18.2 using the same antibody clone and clinically relevant scoring criteria adopted in contemporary therapeutic trials.^2^ Additionally, CLDN18.2 positivity was independently associated with improved overall survival, suggesting that this marker may reflect more favorable tumor biology in addition to serving as a potential therapeutic target. We also demonstrated that CLDN18.2 expression exhibited primary node concordance and was largely preserved in nodal metastases. On the other hand, the intratumoral expression pattern displayed moderate heterogeneity, prompting adequate sampling and standardized evaluation for a reliable and robust assessment.^3,4^

## Future

With CLDN18.2‑targeted regimens already being tested in metastatic PDAC (NCT03816163), future studies should establish standardized pathological assessments of CLDN18.2 while prospectively evaluating its predictive value for CLDN18.2‑targeted therapy. Such efforts may determine whether CLDN18.2 can evolve from a prognostic biomarker into a clinically actionable marker for the perioperative treatment of PDAC.
